# A reverse genetic screen in *Drosophila *using a deletion-inducing mutagen

**DOI:** 10.1186/gb-2004-5-10-r83

**Published:** 2004-09-28

**Authors:** Knud Nairz, Peder Zipperlen, Charles Dearolf, Konrad Basler, Ernst Hafen

**Affiliations:** 1 Zoologisches Institut, Universität Zürich, Winterthurerstrasse 190, Zurich CH-8057, Switzerland; 2 Institut für Molekularbiologie, Universität Zürich, Winterthurerstrasse 190, Zurich CH-8057, Switzerland; 3 Massachusetts General Hospital, Jackson 1402, 55 Fruit St, Boston, MA 02114, USA

## Abstract

We report the use of the cross-linking drug hexamethylphosphoramide (HMPA), which introduces small deletions, as a mutagen suitable for reverse genetics in the model organism *Drosophila melanogaster*. A compatible mutation-detection method based on resolution of PCR fragment-length polymorphisms on standard DNA sequencers is implemented. As the spectrum of HMPA-induced mutations is similar in a variety of organisms, it should be possible to transfer this mutagenesis and detection procedure to other model systems.

## Abstract 2

A new reverse-genetics mutagenesis method that uses the crosslinking drug hexamethylphosphoramide to introduce small deletions has been used to generate and screen pools of mutagenized *Drosophila*, identifying two mutants.

## Background

The fruitfly *Drosophila melanogaster *has been the prime genetic model organism for almost a century. This success story is mainly founded on countless so-called forward genetic screens designed to elucidate gene functions on the basis of their mutant phenotypes. Many of those screens reached a scale that has been termed 'saturating' as they identify all nonredundant genes involved in a certain phenotypic trait. However, forward genetic screens are limited in that they are only capable of uncovering functions that are easily measurable or visible. Furthermore, genes having a redundant or nonessential role are less likely to be found by forward genetics.

The reverse genetic approach to unravel gene function starts with the DNA sequence. Mutations within the gene are induced and identified by various techniques and only subsequently is the mutant phenotype analyzed [1]. Reverse genetics may be undirected or directed, the undirected approach involving random mutagenesis, commonly by transposable elements or by chemicals, the establishment of mutant collections, and the identification of mutations in the gene of interest [2-5]. In contrast, directed reverse genetics is based on techniques that allow for specific inactivation of a gene. These include specific knockdown of gene activities through RNA-mediated interference (RNAi) [6,7] and targeted gene disruption [8,9].

Both undirected and directed reverse genetic techniques have certain advantages and drawbacks. Transposon-based mutagenesis tends to be nonrandom because of the occurrence of hotspots for transposon integration. The use of transposable elements of different origin, such as P-elements and piggyBac, which exhibit a different insertion bias, can partly circumvent this problem. However despite large-scale efforts, the ultimate goal of covering the whole *Drosophila *genome by insertion mutagenesis is far from being achieved [10,11]. Moreover, while null mutants of P-element-tagged genes (P-elements have the tendency to integrate 5' to a gene) can easily be generated by imprecise excision, piggyBac transposons only excise precisely [10].

RNAi and small interfering RNA (siRNA) screens provide a powerful tool to dissect the function of genes at a genome-wide scale [12-14], but the technique is most easily applied to cell cultures and is thus limited to cell-biological problems. Large-scale RNAi screens in multicellular organisms have been done only in *C. elegans *[15] and for technical reasons a similar approach in *Drosophila *is not feasible.

Targeted gene knockout in *Drosophila *allows for generation of both null as well as hypomorphic mutations [16]. However, the technique is time-consuming and technically challenging and hence not applicable on a large scale.

Random mutagenesis in reverse genetics generally relies on well-established techniques and commonly used mutagens, such as ethylmethansulfonate (EMS) [5,17] and *N*-ethyl-*N*-nitrosourea (ENU) [18]. Those chemicals primarily induce single-nucleotide polymorphisms, which can most efficiently be detected by sequencing [19], by denaturing high-pressure liquid chromatography (DHPLC) [5,17], or by enzymatic cleavage of heteroduplex DNA with single-strand-specific endonucleases such as Cel-I [18,20-22]. Mismatch-cleavage analysis and DHPLC require special machinery and DHPLC is not very well suited for high-throughput analysis.

Fast neutrons have also been used to introduce small DNA lesions, which can simply be resolved by agarose electrophoresis after PCR amplification [23]. This kind of mutagenesis may be limited to seeds or to labs in the vicinity of a reactor.

We reasoned that it would be worthwhile to establish a generally applicable reverse genetic technique based on an unbiased and practicable random mutagenesis and an efficient mutation-detection performed on standard laboratory equipment. Here we introduce a novel mutagenesis protocol utilizing the cross-linking drug hexamethylphosphoramide (HMPA) [24], streamlined fly genetics and high-throughput fragment analysis on sequencers to demonstrate the feasibility of our reverse genetics approach.

## Results and discussion

### Fly genetics

There are two ways to handle mutagenized progeny. Either large collections are established and maintained, which then are systematically and continuously screened for mutations of interest, or mutagenized progeny are screened directly and only animals exhibiting a desired trait are kept. The first method is in practice an F3 screen, which requires balancing of mutagenized chromosomes and maintenance of many stocks. This approach is far more labor-intensive than a simple F1 screen of progeny and thus is more suited to stock centers. Moreover, balancer chromosomes have many DNA sequence polymorphisms to wild-type chromosomes (our unpublished data), which will interfere with detection of mutagen-induced sequence polymorphisms.

To circumvent the inherent problems with balancers, we devised an alternative genetic strategy, which had to fulfill the following criteria. First, mutagenized chromosomes have to be passed on in an unrecombined form such that mutations cannot be lost. Second, the mutagenized chromosomes should be brought into an isogenic background for mutation detection. Third, for economic reasons stock-keeping should be kept at an absolute minimum.

**Figure 1:**
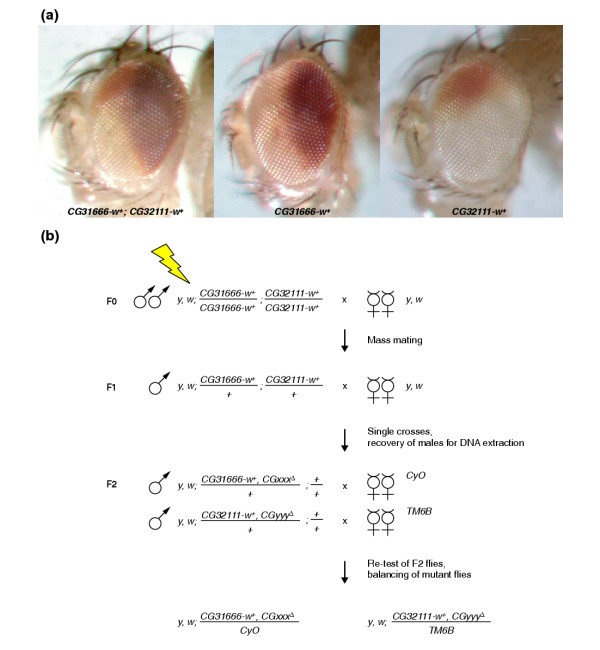
Fly genetics. **(a)** The fly strain used in this study is isogenic to a *yw *standard lab strain, but carries neutral *white*^+ ^transgene insertions on the two major autosomes. The P-element insertion on chromosome 2 localizes to the promoter of *CG31666 *and the other transposon is situated 5' to *CG32111 *on chromosome 3. The *white*^+ ^expression domains are restricted to the anterior and dorsal parts of the eye in the respective strains, and the patterns overlap such that the genotypes can be unambiguously assessed from eye appearance. **(b)** The genetic scheme ensures that mutagenized F0 chromosomes are passed unrecombined, because they are transmitted via males only, and that flies carrying a mutation can easily be singled out on the basis of the eye phenotype. The mutagenized flies are always crossed back to the parental wild type, and only chromosomes from F2 progeny carrying a confirmed mutation are balanced.

We generated a fly strain (KNF306) isogenic to our *yw *wild-type laboratory strain but containing the same dominant marker on the two major autosomes. Both chromosome 2 and chromosome 3 are carrying *white*^+ ^marked P-element insertions, which were chosen because *white*^+ ^expression is restricted to different subregions of the eye (Figure 1a). Chromosome 2 is marked by an insertion in the *CG31666 *locus, which results in *white*^+ ^expression only in the posterior part of the eye. Chromosome 3 harbors an insertion in the promoter of *CG32111*, and this transgene causes dorsal *white*^+ ^expression. The combined expression patterns of both show a 'pie-slice' eye-color appearance (Figure 1a). Thus, the same marker permits us to distinguish between linkage on chromosomes 2 or 3. Neither of the transgenes affects viability, and the line can be kept as a homozygous stock.

Mutagenized chromosomes of strain KNF306 were passaged only via males, which were mated to the parental *yw *strain background. Thus, the marked autosomes remained unrecombined and could be unambiguously assigned because of the dominant character of the *white*^+ ^transgenes (Figure 1b). Mutagen-fed F0 males were mass-mated and F1 males were mated in single crosses (see Materials and methods). After 4 to 5 days, nonsterile F1 males were recovered, pooled in groups of five, and their DNA extracted and analyzed. If a pool gave a positive signal, the crosses were traced back and F2 progeny carrying the mutant chromosome (as judged by the eye-color pattern) of each of the five crosses were individually re-tested. If this re-test was positive, a single F2 male of the respective cross was taken to establish a balanced stock. Non-positive crosses were discarded.

Like any other genomic locus, the *white*^+ ^coding regions of both transgenes constitute targets for mutagenesis, and mutagenic events can be easily scored in the F1 progeny as a loss of the characteristic expression pattern. As discussed later, effectiveness of mutagenesis can be assessed from the occurrence of *white*^- ^progeny, and as an internal control mutation rates at the two loci should be comparable.

The crossing scheme and analysis procedure illustrated was optimized for autosomal genetics. We have generated another strain, KNF307, which in addition carries X chromosomes marked by a characteristic enhancer trap insertion at the *omb *locus (data not shown). However, analysis of X-chromosomal loci would require additional handling of F1 females or mutagenesis of F0 females and hence we did not carry out X-chromosomal screens.

### Mutagenesis

EMS has been used as a deletion-inducing chemical in large-scale screens [25], but unbiased evaluation of its properties suggests that EMS-induced deletions are exceptional [26]. On the other hand, the deletions found by Liu *et al*. [25] ranged in size between 545 base-pairs (bp) and 1,902 bp and would not have been detected by Greene *et al*. [26]. The cross-linking carcinogen hexamethylphosphoramide (HMPA) has been shown to predominately induce deletions that were either in the range 2-315 bp or reached cytologically visible dimensions [24]. As our analysis method restricted the size of PCR fragments to about 800 bp, we chose HMPA as a mutagen, because EMS-induced deletions are likely to affect at least one of the primer-binding sites and would hence be undetectable.

We modified the original HMPA mutagenesis protocol to administer a shorter, but more intense pulse of HMPA ([24], see also Materials and methods). A dose was applied that causes a similar rate of X-linked recessive lethals as standard EMS treatment, but only moderate male sterility (Table 1 and data not shown). We also did not add *N*,*N*-dimethylbenzylamine, which in our hands potentiated the sterilizing activity of HMPA.

It has been reported that F1 progeny may exhibit mosaicism for mutagenized tissue [27]. Mosaic flies could generate a primary positive signal, but might not transmit the mutated gene. We have seen mosaicism at the *white*^+ ^loci and we have found positive F1 pools that did not yield mutant F2 progeny (Table 1 and data not shown). However, we were unable to determine whether some of the primary positives were due to mosaicism or to PCR artifacts.

### Mutation detection

DNA from pools was prepared by a novel high-throughput extraction protocol allowing for up to 2,000 PCRs per pool (see Materials and methods). As HMPA is reported to induce deletions as small as 2 bp and as a mutated allele is diluted 10-fold as a result of our pooling of five flies, we decided to analyze PCR fragments on a sequencer offering maximal resolution and high sensitivity. We have also evaluated the 'poison-primer technique' which is reported to preferentially amplify alleles with a deletion at the poison-primer binding site from large pools [28]. However, the small deletion alleles we have tested did not outperform the amplification of the wild-type allele to the extent previously reported, implicating that the technique is more suited to large deletions and not generally applicable (data not shown).

**Figure 2:**
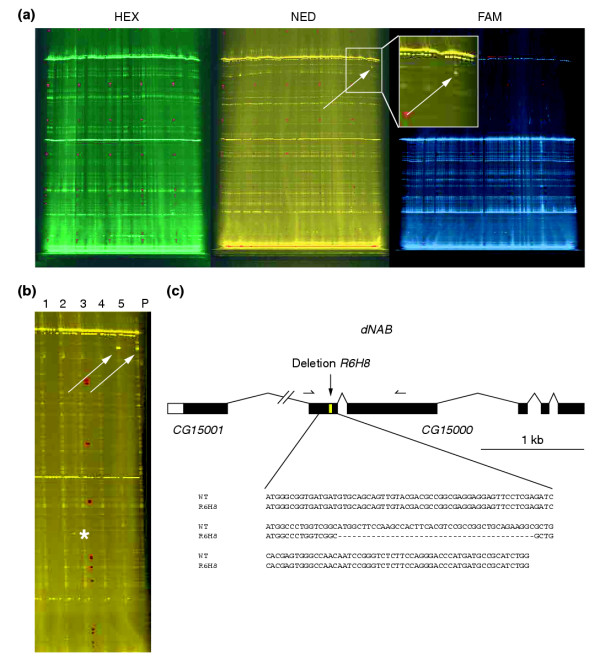
Isolation of a *CG15000/dNAB *null allele. **(a)** Three differently labeled PCR reactions were pooled and concomitantly analyzed on an ABI 377 sequencer. Green, HEX-labeled *white *fragment (control reaction); yellow, NED-labeled *CG15000 *products; blue, FAM-labeled *CG33273/DILP5 *fragment. Color channels are not completely tight and strong products exhibit translucence. Arrows at the screenshot and the magnified inset mark a shorter product specific for one reaction. **(b)** One F2 fly from each cross (1 to 5) of the primary positive pool (P) was analyzed in the context of further F1 screening. Number 5 harbors the deletion allele identified in the pool. The larger, but also specific, fragment in the pool might represent a PCR artifact. The specific band marked by an asterisk turned out to be a PCR artifact as well. **(c)** Genomic organization of wild-type (WT) and mutant *dNAB/CG15000*. Exons are depicted and coding region is symbolized by filled rectangles. Small half arrows indicate primer-binding sites of the PCR-amplified region. The deleted region is filled yellow and the sequence of the wild-type and the mutant alleles starting at an ATG originally annotated as the first codon in the *CG15000 *ORF are aligned below.

PCR products were analyzed on either a gel-based or a capillary sequencer (see Materials and methods). To increase efficiency of mutation detection on gels, we pooled up to three PCR products. These were labeled with different fluorescent tags, partly because they were of similar size (Figure 2a).

### Screening

The efficiency of HMPA mutagenesis could be assessed from the rate of *white*^- ^mutations at the transgenes on chromosomes 2 and 3. Overall, we found 24 mutations in about 62,700 male and female flies. Two flies were mosaic for the mutations. Given that mosaicism can only be scored in eyes and there only in nonoverlapping expression domains, the mutation rates discussed below may be slightly underestimated (Table 1). Male sterility was 25.4 %.

Aguirrezabalaga *et al*. [24] reported a mutation rate of 2.8 × 10^-4 ^at the *vermilion *locus scoring early and late progeny. The rate reached 3.7 × 10^-4 ^when only late progeny was regarded. After a few rounds of screening we have stopped screening early progeny (brood 1 flies, see Materials and methods), because we did not recover any *white*^- ^mutation. As sperm development takes up to 10 days [27], we also consider it unlikely that brood 1 from our crossing scheme will yield appreciable efficiency. Disregarding brood 1, we obtained an average rate of 2.25 × 10^-4 ^mutations at the *white*^+ ^loci, which are about twice as large as the *vermilion *locus. Our mutagenesis procedure involving an overnight incubation with HMPA rather than a 3-day incubation with HMPA and *N*,*N*-dimethylbenzylamine is therefore not much less efficient than the original protocol.

There was no difference in the frequency of induced *white*^- ^mutations between brood 2 and brood 3 (Table 1). The small difference between mutation frequency on the identical *mini-white *genes located on chromosomes 2 and 3 may be attributed to statistical variance, to position effects, to different size of the enhancers driving *white*^+ ^expression or to systemic errors due to the smaller expression domain of the insertion on chromosome 3.

The following additional parameters can be utilized to estimate mutant recovery. The *white *gene for which the mutation rate has been assessed encodes a protein of 688 amino acids from an open reading frame (ORF) of 2,064 bp. We assume that any deletion within the ORF would generate a null phenotype. Only 14 out of 31 HMPA-induced deletions selected at the *vermilion *locus would have been scoreable by our PCR approach, because the remaining 17 mutations were caused by large deletions affecting both primer-binding sites [24].

We designed PCR primers for each gene to be scored such that they encompass the first coding exon and the PCR products are between 450 and 807 bp in size. The average weighted length of our PCR fragments was 710 bp (including two primers of 20 nucleotides each). We thus expect one mutation in 30,317 flies (1/(2.25 × 10^-4 ^× 14/31 × (710 - 2 × 20)/2,064)) or one mutation in 6,063 pools, respectively. Taking into account the fact that two mosaic flies may not have transmitted (reducing the mutation rate to 2.0 × 10^-4^), the estimate would be one mutation in 33,883 flies or one in 6,777 pools.

We have scored 16,902 F1 males at two to 11 loci and recovered two transmitting mutations from about 20,900 analyzed PCR reactions (see Additional data file 2). According to the estimate we would have expected three.

The first mutation detected was a 41-bp deletion in the first exon of *CG15000*, which during the course of this study turned out to be the second exon of the *dNAB *locus (Figure 2c, and see [29]). The deletion causes a frameshift and very probably constitutes a null mutation. As shown in Figure 2a,b, the mutation was identified on a gel-based sequencer in a pool of PCR products labeled with the fluorophore NED (Applied Biosystems) and propagated in one of the five F2 crosses. The mutant chromosome is currently purified by separating the *CG15000/dNAB *allele - easily traceable by a restriction-fragment length polymorphism - from the *white*^+ ^marker (P. Geuking and K.B., unpublished work).

**Figure 3:**
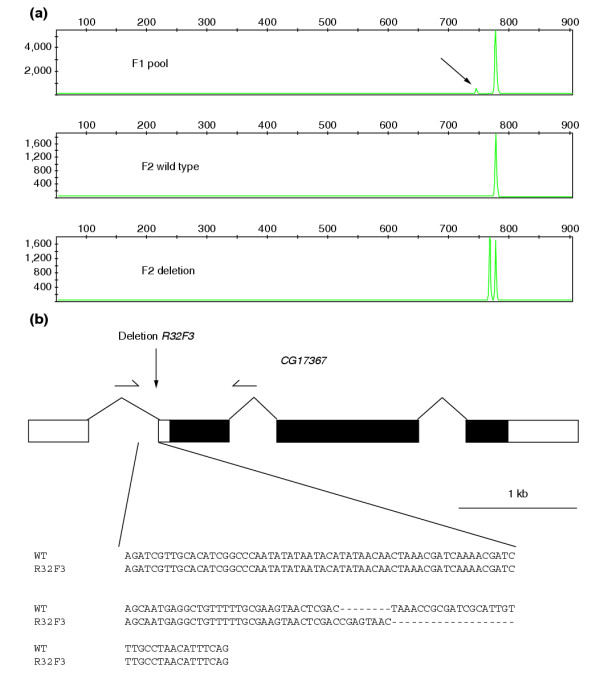
Identification of an HMPA-induced deletion at the *CG17367 *locus. **(a)** Electropherograms of the primary positive pool (top), of one of the four wild-type F2 flies (center), and of a mutant F2 fly (bottom) are shown. The *x*-axis shows fragment size in bp; the *y*-axis, arbitrary intensity units. For unknown reasons the deletion allele runs faster when amplified from pool DNA than from a heterozygous fly. **(b)** Genomic organization of *CG17367/LNK*. Coding (filled rectangles) and noncoding (open) exons are shown and primer-binding sites are indicated by the half arrows. The deletion/insertion is situated 16 bp 5' to a splice site of the first intron, as can be ascertained from the sequence alignment.

Second, we detected a mutation in *CG17367 *on the capillary sequencer (Figure 3a,b). The net 11-bp deletion (19-bp deletion, 8-bp insertion) is situated in the first intron and 5' to the start codon. The allele is viable over a deficiency uncovering the *CG17367 *locus.

This study focused on implementing HMPA mutagenesis for reverse genetics. As discussed above, HMPA efficacy has been assessed from mutations at the *white*^+ ^loci, which have been selected on the basis of phenotype rather than sequence. Thus, our modified HMPA protocol may also prove to be valuable for forward genetic approaches. At the molecular level we could also identify deletions in the *white*^+ ^genes (data not shown), but we have not systematically investigated all of the *white*^- ^mutations.

## Conclusions

While the analysis of PCR fragment-length polymorphisms on our sequencers was very efficient, HMPA mutagenesis turned out to be the limiting parameter. It is about 28-fold less efficient than EMS mutagenesis when it is assumed that all HMPA hits are deleterious (3.2 × 10^-3 ^nucleotide substitutions at the 1 kb *awd *locus [5] for EMS compared to 2.25 × 10^-4 ^deletions per 2 kb *white*^+ ^locus for HMPA), but mutagen dose cannot be increased further because of the concomitant increase in male sterility.

The new techniques that we have introduced increase the diversity of the toolkit available to laboratories interested in conducting reverse genetic screens. The pros and cons of the critical parameters are next considered individually.

### 

#### EMS or ENU versus HMPA as mutagen

HMPA-induced deletions are very likely to induce null mutations when hitting an exon. EMS, on the other hand, primarily induces GC-to-AT transitions, but is not well suited for introducing small deletions. A considerable fraction of the transitions will not affect protein function. In *Arabidopsis*, about 44% of the mutations after EMS mutagenesis were silent, 51% were missense mutations and 5% were nonsense mutations [26]. Similarly, in a zebrafish ENU screen, only 15 out of 270 mutants (5.5%) were truncation mutants [18,19]. Recently, Guo *et al*. [30] determined the tolerance of a protein to random amino-acid changes and determined that about two thirds of amino-acid substitutions were neutral and only 34% were disruptive. Assuming that all truncation mutations are deleterious, it can be concluded that about 22% (34% of 51% plus 5%) of EMS-induced mutations negatively influence protein function. Of those amino-acid substitutions an unknown fraction will retain partial function. Thus, allelic series can be generated through EMS [22] and the generation of partial loss-of-function alleles may be a potential asset of EMS mutagenesis. Overall, HMPA is maximally sixfold less effective at inducing loss-of-function mutations (22% of 28%) than a high dose of EMS, but this disadvantage is compensated for by a more straightforward mutant analysis.

#### Mutant analysis

Mutant analysis depends critically on the mutagen and vice versa. Currently, the most effective way to screen for EMS-induced polymorphisms is the TILLING approach, which, however, requires a second round of PCR, specialized chemistry of the secondary primers, and an enzymatic reaction on the secondary product. TILLING cannot easily be performed on standard sequencers: we have tried to analyze Cel-I cleaved fluorescent heteroduplex DNA on an ABI 3730 sequencer, but did not obtain satisfactory sensitivity (data not shown). HMPA induced mutations can be detected by fragment-length analysis of primary PCR products on standard sequencers. Hence, screening for small deletions reduces PCR costs by a factor of 2 and spares the effort of secondary assays.

#### Mutant handling

Mutant handling is independent of the mutagenesis protocol and may be combined with either EMS or HMPA mutagenesis. For example, TILLING can be performed both on large mutant collections and on a continuous supply of freshly generated mutants.

Finally, given the genotoxic properties of HMPA in both prokaryotes and higher eukaryotes [31,32], both the mutagenesis and the mutation-detection procedures described in this study may be directly transferred to other model organisms.

## Materials and methods

### HMPA mutagenesis

About 150 1-3-day-old F0 KNF306 (*y*, *w*; *CG31666-white*^+^; *CG32111*-*white*^+^) males were starved for 4 to 6 hours in a plastic bottle containing three layers of water-soaked LS14 filter papers (Schleicher & Schüll). A 1.1 ml sample of HMPA solution (5% sucrose, 0.1 M NaPO_4_, 25 mM HMPA, optional 0.05% bromophenol blue) was carefully applied to the filters using a syringe with a long needle (21G2) inserted through the foam stopper. The starved males were exposed to the HMPA solution overnight. Bromophenol blue does not affect mutagenicity detectably, but stains the guts of the flies blue and thus enables mutagen uptake to be monitored and controlled. Freshly eclosed flies do not ingest enough mutagen. HMPA-contaminated plasticware must be disposed of by thermal waste treatment.

### Fly work and crossing procedure

In six bottles containing standard corn medium, each 25 mutagenized KNF306 F0 males (Figure 1a) were allowed to mate to 15 to 20 virgin *yw *females (brood 1). After 2 days males were taken out and crossed to *yw *virgins in new bottles (brood 2A) and this cross was transferred after 3 days (brood 2B). After another 2 days F0 males were recovered and mated to fresh *yw *virgins (brood 3A). F1 males of broods 2A, 2B and 3A were collected and mated individually to three *yw *virgins in about 650 separate crosses per week. Five hundred non-sterile males were removed after 4 to 5 days and five males were pooled for DNA extraction. Fertilized females were returned, and unsuccessful crosses were discarded. If analysis of PCR fragments indicated a primary positive pool, crosses were traced back and kept for further analysis; the other crosses were discarded. From each of the five crosses of primary positive pools a single F2 male or female containing the chromosome of interest as manifested by the typical eye-color pattern was collected for DNA extraction. If PCR analysis yielded a secondary positive result in one of the five F2 flies, a single F2 male containing the chromosome of interest was taken out from the respective cross for balancing (Figure 1b).

The whole crossing scheme requires 6 weeks and was organized such that a mutagenesis was performed every second week (see Additional data file 1).

### Large-scale DNA extraction, PCR and fragment analysis

DNA was extracted in bulk by squishing pools of each five flies through mechanic force in a vibration mill (Retsch MM30) programmed to shake for 20 sec at 20 strokes per second. Flies were placed into wells of a 96-well deep-well plate. Each well was then filled with 500 μl squishing buffer (10 mM Tris-Cl pH 8.2, 1 mM EDTA, 0.2% Triton X-100, 25 mM NaCl, 200 μg/ml freshly added proteinase K) and one tungsten carbide bead (Qiagen). The deep-well plate was then sealed with a rubber mat (Eppendorf) and clamped into the vibration mill. (Tungsten carbide beads can be recycled: after an overnight incubation in 0.1 M HCl and thorough washing in double-distilled water (ddH_2_O) the beads were virtually free of contaminating DNA.) Debris was allowed to settle for about 5 min and each 50 to 100 μl of supernatant were transferred into a 96-well PCR plate. The reactions were incubated in a thermocycler for 30 min at 37°C, and finally for 5 min at 95°C to heat-inactivate proteinase K.

A Tecan pipeting robot was used for PCR setup. To 5 μl of template DNA, master-mix was added and PCR was performed on an MJR thermocycler that was integrated into the robot. The master-mix per reaction was composed of 20.48 μl ddH_2_O, 0.6 μl MgCl_2 _(25 mM), 0.6 μl dNTPs (10 mM), 0.1 μl fluorescently labeled primer 1 (100 μM), 0.1 μl primer 2 (100 μM), 0.12 μl hot-start Taq polymerase (HotStar, Qiagen, 5 U/μl), 3 μl 10× buffer containing MgCl_2 _(Qiagen). Cycling conditions were 95°C 15 min, 35 × (95°C 20 sec, 60°C 30 sec, 72°C 1 min), 72°C 2 min, 4°C.

Three differently labeled PCR reactions (oligos were 5' labeled with Applied Biosystems' fluorophors FAM, NED and VIC, respectively) were then pooled. To facilitate sizing of fragments we also added ROX1000 size marker (Applied Biosystems) to five DNA pools. Samples of 1.5 μl pooled DNA were mixed with 1.5 μl loading buffer (consisting of one part 25 mM EDTA pH 8.0 with 50 mg/ml blue dextran and five parts HiDi formamide (Applied Biosystems)). The reactions were incubated for 3 min at 95°C, cooled down, and 1.5 μl each were loaded onto a 96-lane ABI 377 sequencer. Run conditions were as follows: 1 h pre-run at 1,000 V, 35 mA, 51°C and 10 h run at 2,400 V, 50 mA, 51°C. Gel images recorded at four different color channels by the GeneScan software were analyzed visually.

Slight modifications to this protocol were introduced for analysis performed on an ABI 3730 capillary sequencer. First, DNA was diluted 20-fold before PCR. Second, after PCR, reactions were diluted 100-fold and 2 μl of diluted PCR products were added to each 15 μl HiDi formamide (Applied Biosystems). PCR product was diluted on a Tecan pipeting robot. Diluted DNA was denatured for 2 min at 95°C before analysis. Sample injection (10 sec) and analysis (12,000 scans) was done according to standard protocols. Identification of deletion fragments was then performed by visual inspection of gel-images generated by the Data Collection Software (Array Viewer option, Applied Biosystems). No internal size standard was used, as deletion fragments were identified relative to wild-type PCR product.

## Additional data files

The following additional files are available with the online version of this paper. Additional data file 1 contains the time schedule of mutagenesis, fly work, and screening. The whole procedure takes six weeks and is organized such that one mutagenesis has to be performed every second week to generate a continuous supply of mutagenized progeny. Additional data file 2 contains information on the 10 other genes scored. Gene names, fluorescent labels, fragment lengths and the number of analyzed F1 flies are given. Labeled primers were ordered from Applied Biosystems. Primer sequences are available upon request.

## Supplementary Material

Additional data file 1 The time schedule of mutagenesis, fly work, and screening Click here for additional data file

Additional data file 2 Information on the 10 other genes scored Click here for additional data file

## Figures and Tables

**Table 1:** Mutation rates induced by HMPA treatment as assessed from *white*^- ^mutations at the transgene insertions on chromosomes 2 and 3

*white*^+ ^loci	Brood	Mutation rate (× 10^-4^)	Mosaicism at *white*^+ ^loci
Both loci	2A + 2B and 3A	2.25	
On chromosome 2	2A + 2B and 3A	2.68	1/14
On chromosome 3	2A + 2B and 3A	2.0	1/10
Both loci	2A + 2B	2.35	
Both loci	3A	2.33	

Numbers are based on 19 mutations recovered after screening of brood 1 has ceased. Two out of a total of 24 *white*^- ^mutants (five mutants were recovered while brood 1 was screened) were mosaic for the mutation. Mutations on chromosome 2 were slightly more common than mutations on chromosome 3; brood-2- and brood-3-derived mutations were equally frequent.
