# Supplementary material for: A reverse genetic screen in Drosophila using a deletion-inducing mutagen
Source: Genome Biol. 2004 Sep 28;5(10):R83. doi: 10.1186/gb-2004-5-10-r83 (PMC545603; doi:10.1186/gb-2004-5-10-r83)
Supplement: Additional data file 1 — The time schedule of mutagenesis, fly work, and screening [file gb-2004-5-10-r83-s1.doc]

**Supplementary table 1:** Time schedule of mutagenesis, fly work, and screening

| week 1 | | | | | | | week 2 | | | | | | | week 3 | | | | | | | week 4 | | | | | | | week 5 | | | | | | | week 6 | | | | | | |
| --- | --- | --- | --- | --- | --- | --- | --- | --- | --- | --- | --- | --- | --- | --- | --- | --- | --- | --- | --- | --- | --- | --- | --- | --- | --- | --- | --- | --- | --- | --- | --- | --- | --- | --- | --- | --- | --- | --- | --- | --- | --- |
| Mo | Tue | We | Thu | Fri | Sat | Sun | Mo | Tue | We | Thu | Fri | Sat | Sun | Mo | Tue | We | Thu | Fri | Sat | Sun | Mo | Tue | We | Thu | Fri | Sat | Sun | Mo | Tue | We | Thu | Fri | Sat | Sun | Mo | Tue | We | Thu | Fri | Sat | Sun |
|  | 1 | 2 |  |  |  |  |  |  |  |  |  |  |  |  |  |  |  |  |  |  |  |  |  |  |  |  |  |  |  |  |  |  |  |  |  |  |  |  |  |  |  |
|  |  |  |  | 3 |  |  |  |  |  |  |  |  |  |  |  |  |  |  |  |  |  |  |  |  |  |  |  |  |  |  |  |  |  |  |  |  |  |  |  |  |  |
|  |  |  |  |  |  |  | 4 |  |  |  |  |  |  |  |  |  |  |  |  |  |  |  |  |  |  |  |  |  |  |  |  |  |  |  |  |  |  |  |  |  |  |
|  |  |  |  |  |  |  |  |  | 5 | (5) |  |  |  |  |  |  |  |  |  |  |  |  |  |  |  |  |  |  |  |  |  |  |  |  |  |  |  |  |  |  |  |
|  |  |  |  |  |  |  |  |  |  |  |  |  |  |  |  | 6 | (6) |  |  |  |  |  |  |  |  |  |  |  |  |  |  |  |  |  |  |  |  |  |  |  |  |
|  |  |  |  |  |  |  |  |  |  |  |  |  |  |  |  |  |  |  |  |  |  |  | 7 | (7) |  |  |  |  |  |  |  |  |  |  |  |  |  |  |  |  |  |
|  |  |  |  |  |  |  |  |  |  |  |  |  |  |  |  |  |  |  |  |  | 8 | 9 | 10 |  |  |  |  | 11 | 12 | 13 |  |  |  |  |  |  |  |  |  |  |  |
|  |  |  |  |  |  |  |  |  |  |  |  |  |  |  |  |  |  |  |  |  |  |  |  |  |  |  |  | 14 | 15 | 16 |  |  |  |  | 17 | 18 | 19 |  |  |  |  |

1 Mutagenesis of F_0_ males

2 F_0_ cross - brood 1: 25 males and 15-20 virgins each in 6 bottles, females and bottles will be discarded after 2 days

3 F_0_ cross - brood 2A: F_0_ males recovered from brood 1 and mated to fresh virgins in new bottles

4 Transfer of brood 2A into new bottles (brood 2B)

5 F_0_ cross - brood 3A: F_0_ males recovered from brood 2B and mated to fresh virgins in new bottles

6 F_1_ cross: male progeny of brood 2A crossed to each 3 virgins in plastic tubes

7 F_1_ cross: male progeny of brood 2B and 3A crossed to each 3 virgins each in plastic tubes

8 DNA-extraction from pools of each five F_1_ males recovered from F_1_ cross (6)

9 PCR and dilution of PCR reactions

10 Analysis of PCR products

11 DNA-extraction from F_2_ flies from primary positive pools

12 PCR, dilution, and analysis

13 Establishment of a mutant strain with one F_2_ male from the positive tube

14 DNA-extraction from pools of each five F_1_ males recovered from F_1_ cross (7)

15 PCR and dilution of PCR reactions

16 Analysis of PCR products

17 DNA-extraction from F_2_ flies from primary positive pools

18 PCR, dilution, and analysis

19 Establishment of a mutant strain with one F_2_ male from the positive tube
