# Supplementary material for: A reverse genetic screen in Drosophila using a deletion-inducing mutagen
Source: Genome Biol. 2004 Sep 28;5(10):R83. doi: 10.1186/gb-2004-5-10-r83 (PMC545603; doi:10.1186/gb-2004-5-10-r83)
Supplement: Additional data file 2 — Information on the 10 other genes scored [file gb-2004-5-10-r83-s2.doc]

| gene | label | fragment length (bp) | number of flies screened (in pools of five) |
| --- | --- | --- | --- |
| CG15000 | NED | 798 | 1390 |
| *CG17367/LNK* | NED | 801 | 8702 |
| *CG33273/DILP5* | FAM | 609 (466) | 16902 |
| *CG5110/MP1* | NED | 679 (712) | 14082 |
| *CG8167/Dilp2* | VIC | 733 | 13652 |
| *CG5484* | NED | 790 | 8702 |
| *CG9138* | FAM | 755 | 8702 |
| *CG5475/p38a* | VIC | 708 | 8702 |
| *CG3994* | FAM | 740 | 8702 |
| *CG9648/dmax* | FAM | 746 | 8397 |
| *CG7393/p38b* | FAM | 807 | 6569 |
| *white* | HEX | 798 |  |

Supplementary table 2: Apart from *CG15000* and *CG17367*, ten other genes were scored. Gene names, fluorescent labels, fragment lengths, and the number of investigated F_1_ flies are given.

Labeled primers were ordered from Applied Biosystems. Primer sequences are available upon request.
